# hERG1 channel expression associates with molecular subtypes and prognosis in breast cancer

**DOI:** 10.1186/s12935-018-0592-1

**Published:** 2018-07-05

**Authors:** Jessica Iorio, Icro Meattini, Simonetta Bianchi, Marco Bernini, Virginia Maragna, Luca Dominici, Donato Casella, Vania Vezzosi, Lorenzo Orzalesi, Jacopo Nori, Lorenzo Livi, Annarosa Arcangeli, Elena Lastraioli

**Affiliations:** 1 Department of Experimental and Clinical Medicine, Section of Internal Medicine, University of Florence, Viale GB Morgagni, 50, 50134 Florence, Italy; 2 Doctorate Course in Genetics, Oncology and Clinical Medicine, University of Siena, Siena, Italy; 3 Radiation Oncology Unit, Department of Oncology, University of Florence, Azienda Ospedaliero-Universitaria Careggi, Florence, Italy; 4 Section of Pathological Anatomy, Department of Surgery and Translational Medicine, University of Florence-Azienda Ospedaliero-Universitaria Careggi, Florence, Italy; 5 Breast Unit Surgery, Department of Oncology, Azienda Ospedaliero-Universitaria Careggi, Florence, Italy; 6 Diagnostic Senology Unit, University of Florence, Azienda Ospedaliero-Universitaria Careggi, Florence, Italy

**Keywords:** hERG1, Potassium channels, Breast cancer, Molecular subtype, Immunohistochemistry

## Abstract

**Background:**

Breast cancer (BC) is the most frequent malignancy among females worldwide. Despite several efforts and improvements in early diagnosis and treatment, there are still tumors characterized by an aggressive behavior due to unfavorable biology, thus quite difficult to treat. In this view, searching for novel potential biomarkers is mandatory. Among them, in the recent years data have been gathered addressing ion channel as important players in oncology.

**Methods:**

A retrospective pilot study was performed on 40 BC samples by means of immunohistochemistry in order to evaluate hERG1 potassium channels expression in BC.

**Results:**

We provide evidence that hERG1 is expressed in all the BC samples analyzed. hERG1 expression was significantly associated with molecular subtype with the highest expression in Luminal A and the lowest in basal-like tumors (p = 0.001), tumor grading (the highest hERG1 expression in well-moderate differentiated tumors, p = 0.020), estrogen receptors (high hERG1 expression in ER-positive samples, p = 0.008) and Ki67 proliferative index (high hERG1 scoring in samples with low proliferative index, p = 0.038). Also, a p value close to significance was noticed for the association between hERG1 and HER2 expression (p = 0.079). At the survival analysis, patients with high hERG1 expression turned out to have a longer progression-free survival, although statistical significance was not reached (p = 0.195). The same trend was observed analyzing local relapse free-survival (LRFS) and metastases-free survival (MFS): patients with higher hERG1 scoring had longer LRFS and MFS (p = 0.124 and p = 0.071, respectively).

**Conclusions:**

The results of this pilot study provide the first evidence that the hERG1 protein is expressed in primary BC, and its expression associates with molecular subtype. hERG1 apparently behaves as a protective factor, since it contributes to identify a subset of patients with better outcome. Overall, these data suggest that hERG1 might be an additional tool for the management of BC, nevertheless further investigations are warranted to better clarify hERG1 role and clinical usefulness in BC.

## Background

Breast cancer (BC) is the most frequent malignancy among females worldwide [1]. Unfortunately, its incidence is still increasing, particularly in developing countries [2, 3] and it has been predicted to keep growing at least until 2050 [4]. Although 1 in 8 women can experience this fearful event in their lifetime in Western countries [5], mortality has been decreasing [1, 6]. Screening protocols, early diagnosis, surgery and adjuvant therapy (chemotherapy, biological therapies and radiotherapy) have greatly contributed to this achievement. A key to success in this battle has also been the understanding of the biological nature of BC. Nowadays, not only the TNM stage but also the identification of the biological subtype is crucial in the clinical management of BC. The use of endocrine therapy, chemotherapy drugs regimens, monoclonal antibodies, and kinase inhibitors are mostly driven by specific biomarkers. Such biomarkers can easily be defined by immunohistochemistry (IHC). In particular, expression of estrogen receptor (ER), progesterone receptor (PgR), Ki67 proliferative index, and HER2 status, form the basis of the most commonly used four pathological subtypes. Such a classification scheme is convenient and helpful in guiding clinicians to choose appropriate therapy options [7]. However, several concerns still remain in the clinical management of BC. First, BC is still misdiagnosed in around 4% of cases [8]. Second, there is no target therapy for the cases of basal-like BC. Third, systemic therapies often have undesirable side effects and limited time of effectiveness due to onset of drug resistance [9]. Consequently, additional functional biomarkers are strongly needed.

In the last 20 years, ion channels have been proven to be novel biomarkers in cancer (reviewed in [10]), as well as novel targets for cancer therapy, due to their easy druggability [11]. Among ion channels expressed in BC, particular attention has been focused on K^+^ channels, both voltage dependent (Kv10.1, Kv1.3, hERG1) and Ca^2+^-activated (KCa1.1, KCa2.1, KCa2.2, KCa2.3, KCa3.1). All these channels are overexpressed in primary BC and cell lines, and several correlations with clinico-pathological features have been demonstrated. In particular, in primary samples it was shown that KCa1.1 channels are positively correlated to ER expression [12], the occurrence of brain metastases [13], high stage, nuclear grade, proliferation and poor prognosis [14].

Among ion channels dysregulated in cancer, the voltage-gated K^+^ channel hERG1 was shown to be overexpressed in neoplastic cell lines and human primary tumors (reviewed in [15]). hERG1 overexpression was demonstrated in many solid cancers, from esophageal [16, 17] to gastric [18] and colorectal cancers [19–22], and several associations with clinico-pathological parameters and outcome were shown. As regards BC, most of the available evidences for K^+^ channels come from in vitro studies. hERG1 has been shown to induce cell senescence [23] as well as to mediate the transcription of p21^waf/cip^ in BC cells [24]. Although the analysis of public datasets reported the overexpression of the hERG1 encoding gene (*KCNH2*) in BC [25], no studies on primary BC reporting clinical correlations according to hERG1 protein expression have been published so far.

In the present paper we evaluated the IHC-based hERG1 expression profile in a cohort of BC specimens and analyzed associations with molecular and clinico-pathological features.

## Materials and methods

### Retrospective study on human samples

#### Pathological evaluation

Every case of BC was diagnosed by two breast pathologists (SB, VV). Cancer molecular subtype was determined by ER, PgR and HER2 status, according to the 15th St. Gallen International Breast Cancer Conference, 2017, as follows:

1. Luminal A: ER+ and/or PgR+, HER2−, low Ki67. Luminal A tumors are generally low-grade, grow slowly and have a favorable prognosis;
2. Luminal B: ER+ and/or PgR+, HER2−, high Ki67 or ER+ and/or PgR+, HER2+, any Ki67. Luminal B tumors grow faster than Luminal A and have a worse prognosis;
3. HER2-enriched: ER−, PgR−, HER2+. HER2-enriched tumors grow faster than luminal cancers and generally have a worse prognosis, although they can be treated with targeted therapies directed against the HER2 protein (trastuzumab, pertuzumab, lapatinib and T-DM1 or ado-trastuzumab emtansine).
4. Basal-like (also known as “triple negative”): ER−, PgR−, HER2−. Basal-like tumors are characterized by the worst prognosis since they are the hardest to treat.

Hormone receptor status was reported as negative when < 1% of tumors cells stained at IHC. HER2 status was determined only by IHC in cases scored as 0 or 1+ (negative) and 3+ (positive). Fluorescence in situ hybridization (FISH) was used in 2+ cases.

#### Sample collection

A retrospective study was carried out on a set of 40 BC samples (belonging to patients with median age 53.5 years, range 28–87 years) of different pathological subtype collected from the archives of the Section of Pathological Anatomy, Department of Surgery and Translational Medicine, University of Florence-Azienda Ospedaliero-Universitaria Careggi, Florence, after informed written consent. All the samples belonging to Luminal B subtype were HER2 positive. Patients underwent surgery at Breast Surgery Unit, Department of Oncology, Azienda Ospedaliero-Universitaria Careggi, Florence. Data concerning follow up were retrieved from the database of the Radiation Oncology Unit, Department of Oncology-University of Florence, Azienda Ospedaliero-Universitaria Careggi, Florence, where takes place patients clinical follow up. In particular, the following parameters were taken into account: presence of local relapse, presence of distant metastases, progression of the disease, survival.

#### IHC and scoring of the staining

IHC was performed on formalin-fixed, paraffin-embedded samples using the anti-hERG1 monoclonal antibody (Dival Toscana Srl) at a final dilution 1:500 following the protocol already published—by Lastraioli E et al. [21].

Immunostaining was carried out with a commercially available kit (PicTure-Max polymer Detection kit, Invitrogen) according to manufacturer’s instructions. hERG1 expression was evaluated by an IHC-based score obtained through the combination of the estimate of the percentage of immunoreactive cells (quantity score) with the estimate of staining intensity (staining intensity score). Staining intensity was rated on a scale of 0–3, with 0 = negative; 1 = weak; 2 = moderate, and 3 = strong. The raw data were converted to the complete score by multiplying the “quantity” and “staining intensity” scores. The combined score was as follows: “Score 0”: total score = 0; “Score 1”: total score = 1–100; “Score 2”: total score = 101–200; “Score 3”: total score = 201–300. Samples were evaluated by two independent investigators (SB and EL).

### Statistical analysis

The presence of association between demographic, clinical and biological characteristics as well as the association between biomarkers’ expression was evaluated by Fisher’s exact test (two-tailed, p < 0.05). The Pearson correlation coefficient (R) was calculated to evaluate relationships between continuous variables (R = − 1 negative relationship, R = 0 no relationship, R = 1 positive relationship). Survival analyses were performed applying Log Rank Test and Kaplan–Meier plots. Statistical analyses were performed using Stata 9.1 (StataCorp, TX, USA) and Microcal Origin 9.0 (OriginLab, MA, USA).

## Results

### hERG1 is expressed in BC primary samples

**Fig. 1:**
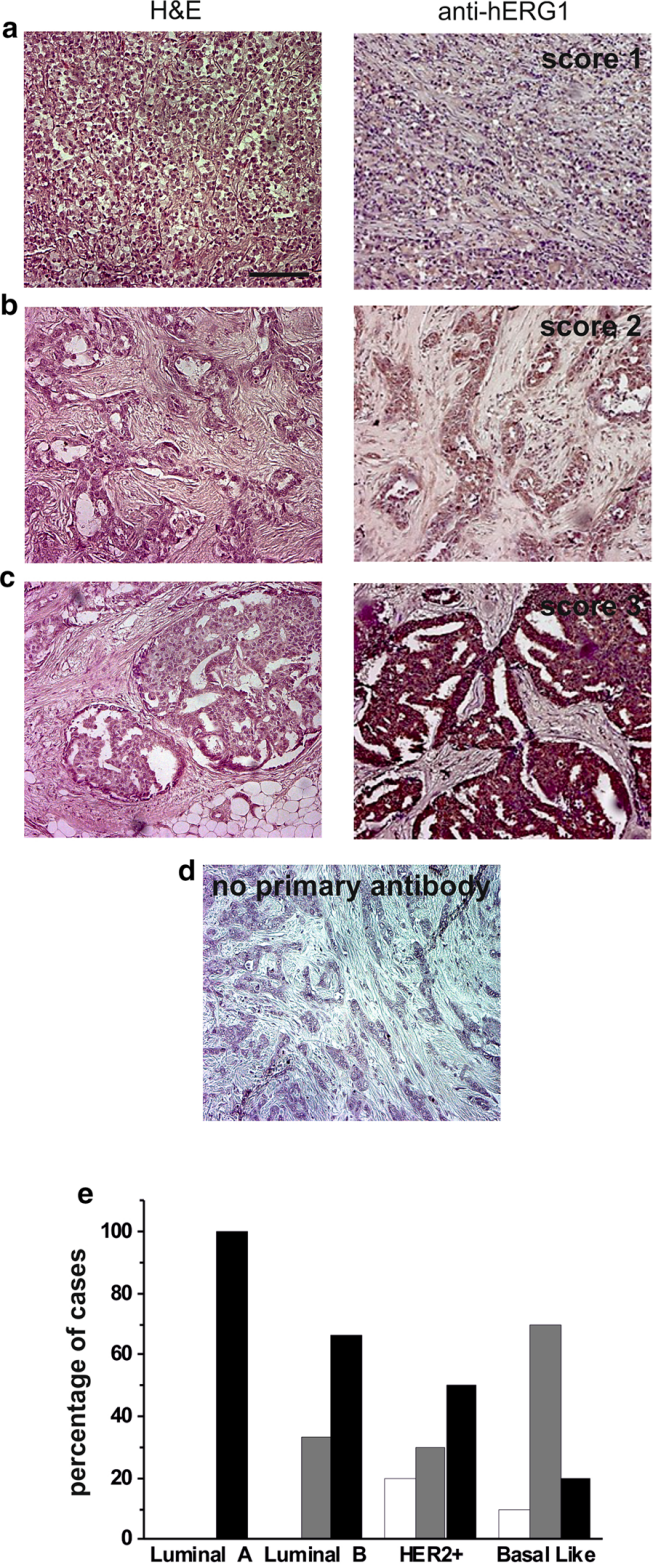
IHC and scoring system assessment for hERG1 in the different molecular subtypes. **a**–**c** Hematoxylin–Eosin staining (right panels) and IHC (left panels) of representative samples. **a** Score 1. **b** Score 2. Score 3 (**d**). No primary antibody (negative control). IHC procedures and scoring assessment were performed according to what reported in the “Materials and methods” section. Original magnification ×200. Scale bar: 100 μm. **e** Histogram summarizing hERG1 levels of expression in the four different pathological subtypes of breast cancer evaluated by IHC. White bars: Score 1, Grey bars: Score 2, Black bars: Score 3

Forty primary BC samples were retrospectively studied by IHC to assess hERG1 expression. Overall, hERG1 was expressed in 100% of BC samples. According to the scoring system applied (see “Materials and methods”), 7.5% of the samples were scored as Score 1, 30.0% showed a moderate hERG1 expression (Score 2) and 57.5% had a very high hERG1 expression (Score 3). Representative pictures of the IHC performed with the anti-hERG1 monoclonal antibody (see “Materials and methods”), relative to each score, are reported in Fig. 1 together with the corresponding Hematoxylin–Eosin microphotographs.

Dividing the samples according to molecular subtype, it emerged that hERG1 scoring was higher in Luminal A and B samples (100 and 66.7%, respectively, of the samples were scored as “3”) with respect to HER2+ and basal-like samples, in which 50 and 20% of the samples were scored as “3”(Fig. 1d).

### Associations with molecular and clinico-pathological parameters

Data gathered from IHC experiments were analyzed through Fisher Exact test and several associations emerged (Table 1). Interestingly, hERG1 scoring was associated with molecular subtype (p = 0.001) being higher in Luminal A, progressively decreasing in Luminal B, HER2+ and basal-like tumors. We also found an association with grading, with higher scoring in G1 and G2 samples (p = 0.020). Moreover, hERG1 scoring was significantly associated with ER expression, with higher hERG1 scoring in ER-positive samples (p = 0.008), and inversely associated with Ki67 (higher hERG1 scoring in samples with Ki67 index lower to 15%, p = 0.038). Finally, an inverse association with HER2 expression emerged, with the highest hERG1 expression in HER2 negative samples, whose p value (0.079) was close to be significant. Since in the HER2+ group (composed of samples with HER2 scoring = 3) hERG1 expression was unequally distributed (see Table 1), we analyzed the percentage of positive cells per microscopic field applying the Pearson correlation coefficient and we found that the correlation between the two proteins was weak (R = 0.118), in accordance with the results of Fisher’s Exact Test (p = 0.079).

**Table 1:** Associations between hERG1 expression and molecular and clinicopathological parameters

	hERG1 score 0	hERG1 score 1	hERG1 score 2	hERG1 score 3	p value
Molecular subtype					
Luminal A	0 (0%)	0 (0%)	0 (0%)	11 (100%)	0.001*
Luminal B	0 (0%)	0 (0%)	3 (33.3%)	6 (66.7%)	
HER2+	0 (0%)	2 (20%)	3 (30%)	5 (50%)	
Basal-like	0 (0%)	1 (10%)	7 (70%)	2 (20%)	
Grading					
G1	0 (0%)	0 (0%)	0 (0%)	6 (100%)	0.020*
G2	0 (0%)	0 (0%)	0 (0%)	6 (100%)	
G3	0 (0%)	3 (10.7%)	13 (46.4%)	12 (42.9%)	
TNM stage					
I	0 (0%)	1 (4.3%)	7 (30.4%)	15 (65.2%)	0.071
II	0 (0%)	1 (11.1%)	5 (55.6%)	3 (33.3%)	
III	0 (0%)	0 (0%)	1 (14.3%)	6 (85.7%)	
IV	0 (0%)	1 (100%)	0 (0%)	0 (0%)	
ER					
Negative	0 (0%)	3 (15.8%)	9 (47.4%)	7 (36.8%)	0.008*
Positive	0 (0%)	0 (0%)	4 (19.1%)	17 (80.9%)	
PgR					
Negative	0 (0%)	3 (12.5%)	10 (41.7%)	11 (45.8%)	0.083
Positive	0 (0%)	0 (0%)	3 (18.7%)	13 (81.2%)	
HER2					
Negative	0 (0%)	1 (7.7%)	7 (53.8%)	5 (38.5%)	0.079
Score 1	0 (0%)	0 (0%)	0 (0%)	7 (100%)	
Score 2	0 (0%)	0 (0%)	0 (0%)	0 (0%)	
Score 3	0 (0%)	2 (10%)	6 (30%)	12 (60%)	
Ki67					
≤15%	0 (0%)	0 (0%)	0 (0%)	8 (100%)	0.038*
>15%	0 (0%)	3 (9.4%)	13 (40.6%)	16 (50%)	

* p < 0.05 (Fisher Exact Test)

### Survival analyses

Since the study was retrospective, all the patients had a long follow up. Overall Survival was not performed, since all the patients were alive at the end of the study. Thus, we performed survival analyses to evaluate progression-free survival (PFS), local relapse-free survival (LRFS) and distant metastases-free survival (DMFS). From such analyses it emerged that patients with high hERG1 expression had a longer LRFS and PFS, although statistical significance was not reached (p = 0.124 and p = 0.195, respectively). When performing a survival analysis taking into account metastases-free survival (MFS) it emerged a similar trend, since patients with higher hERG1 scoring had longer MFS, with a p value close to the significance (p = 0.071).

**Fig. 2:**
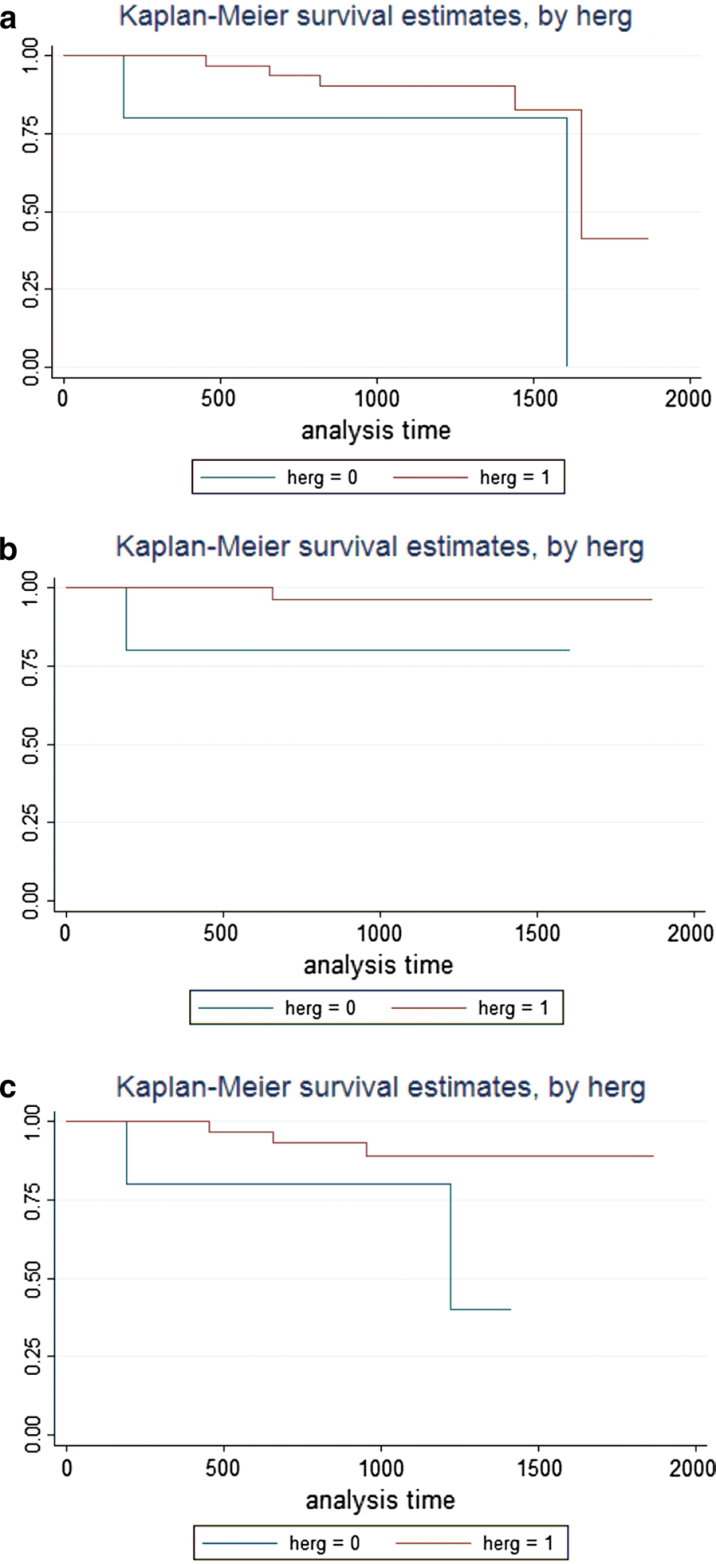
Kaplan Meier plots according to hERG1 scoring. **a** PFS; **b** LRFS; **c** DMFS. Blue curves: hERG1 negative samples (Score ≤ 150), red curves: hERG1 positive samples (Score > 150)

Kaplan–Meier plots of PFS, LRFS and DMFS according to hERG1 scoring are in Fig. 2.

## Discussion

In the present paper we provide evidence that hERG1 potassium channels are expressed in BC and positively affect patients’ prognosis. Data presented here are the results of a pilot study performed on 40 BC primary samples from which we showed that hERG1 protein is expressed in a high percentage of samples belonging to all the four molecular subtypes. To our knowledge this is the first demonstration of the association of hERG1 channels with BC molecular subtypes. These findings open new perspectives for BC management, since basal-like BC include most of triple negative BC, that do not express ER, PgR and HER2 being therefore the hardest to treat: in this scenario, hERG1 presence might be helpful for targeted therapies. When dealing with basal-like BC, it is worth noting that several subgroups have been identified, taking into account genomic instability and rearrangements (reviewed in [26]), and it gets the management of such disease more complicated. To ensure the best treatment option for each patient, the concept of “personalized” or “tailored” therapies was particularly emphasized at the 2017 St. Gallen International Expert Consensus.

Also more interestingly, we showed here that hERG1 is highly expressed in samples characterized by positive prognostic features such as Luminal A molecular subtype, well and moderately differentiated tumors (G1 and G2), low proliferation rate (Ki67 index ≤ 15%). Also, a borderline association was noticed when analyzing HER2 scoring, with the highest hERG1 expression in HER2 negative samples (characterized by better prognosis). Overall, these findings are in accordance with the results we obtained from survival analyses and a trend emerged addressing hERG1 as a protective factor, positively affecting BC in terms of PFS, LRFS and DMFS. The data we gathered from the present study fit well in the context described in a paper published in 2013 [27], in which an “IC30 gene signature” composed of 30 ion channel genes was defined and proven to be a prognostic marker in BC, independently of clinical and pathological prognostic factors.

Our findings might add an element to the complex picture of BC, delineated over the last years. In fact, even using extensive genomic profiling four coherent groups similar to the intrinsic subtypes emerge as stated in the 2015 St. Gallen Consensus Conference [28]. From this perspective the greatest controversies arise in using or not chemotherapeutic agents in “luminal” cases with hormones receptors positivity and negative HER2, which can display very different clinical behaviors. Another group of patients needing a deeper investigation is indeed the triple-negative. Again with the St. Gallen 2015 panel members’ words, we could state that “further dissection of subtypes within triple-negative breast cancer reveals seven distinct groupings, which differ markedly in their clinical response to neoadjuvant chemotherapy [29]. Preclinical studies also show heterogeneity of response to other agents in cell lines of the different triple-negative subtypes [30]”. In both these two groups, genotyping has partially answered to the need of this further characterization [26, 31] and nowadays several commercial kits (i.e. ONCOTYPE DX^®^, MAMMAPRINT^®^, BLUEPRINT^®^, TARGETPRINT^®^, PROSIGNA^®^) that can be used to analyze a different number of genes set and better define the biological essence of every single case are available. Knowledge of specific genes and their products, implicated in BC tumorigenesis, has proved to be of utmost importance in BC cure.

The clinical utility of the addition of the 70-gene signature test (MammaPrint^®^) to standard clinical-pathological criteria in selecting patients for adjuvant chemotherapy was largely demonstrated by the recently published MINDACT trial (ClinicalTrials.gov number, NCT00433589; EudraCT number, 2005-002625-31). This randomized, phase 3 study, evaluated different adjuvant approaches on 6693 women affected by early-stage breast cancer basing on their genomic (using the 70-gene signature) and clinical risk (using a modified version of Adjuvant! Online). Among women at high clinical risk and low genomic risk for recurrence, the receipt of no chemotherapy on the basis of the 70-gene signature led to a 5-year rate of survival without distant metastasis that was 1.5% points lower than the rate with chemotherapy, showing that around 46% of women with breast cancer who are at high clinical risk might not require chemotherapy [32].

Unfortunately, this is an expensive task, which cannot be reproduced on a routinely basis in most Public Health Systems. Therefore, the search for surrogate biomolecular markers is definitely worth to be carried on in the BC scenario, since this can translate is useful prognostic and therapeutic tools for clinicians. Among biomolecular markers, stemness markers represent a novel and interesting tool for the management of different kind of tumors [33]. In BC it was shown that cancer stem cells correlate with disease progression and prognosis in in vivo models [34]. More recently, Finicelli et al. [35] demonstrated that the stem cell marker SOX2 is an independent factor to predict early recurrence in BC.

A recently published review [36] summarized the identified biomarkers of TNBC that comprise basal-like BC, although the two categories are not exactly identical and overlapping. Some molecules are overexpressed in TNBC, although not exclusive of this subgroup of BC, and could therefore serve as biomarkers for this subgroup of BC (i.e. EGFR, Ki67, VEGF-A, p53). In particular, it was shown that EGFR expression is related to the aggressiveness of the disease and poor response to chemotherapy [37], and it was proposed that EGFR might be used for the differential classification of basal-like BC. These findings are of particular interest within our research field, since we demonstrated that hERG1 channel expression is significantly associated with EGFR expression in pancreatic ductal adenocarcinomas [38] and colorectal cancers [21]. This association might be exploited also for therapy purposes, since EGFR is a target of cetuximab that might be used in combination with specific anti-hERG1 drugs. A similar approach might be applied to VEGF-A whose high expression is associated with poor prognosis [36]: in fact, we showed that in colorectal [21] and gastric [18] cancers hERG1 expression is significantly associated with VEGF-A expression and the combined therapy with bevacizumab and hERG1 blockers impairs tumor growth in mouse models [18]. More recently it was also shown that hERG1 interaction with β1 integrins mediates BC metastatization in immunodeficient mice [39].

## Conclusion

Data reported in the present paper, although preliminary, open new promising perspectives for BC management, and the inhibitors of the channels might be used for combined therapy together with EGFR and VEGF-A blockers. The results of this pilot study indicate that hERG1 expression is associated with clinical-pathological features in BC and it behaves as a positive factor thus it might be an additional tool for the management of BC. Nevertheless, further investigations are warranted to better clarify hERG1 role and usefulness in BC.
